# Common origin of ornithine–urea cycle in opisthokonts and stramenopiles

**DOI:** 10.1038/s41598-020-73715-8

**Published:** 2020-10-07

**Authors:** Aleš Horák, Andrew E. Allen, Miroslav Oborník

**Affiliations:** 1 Biology Centre, Czech Academy of Sciences, Institute of Parasitology, Branišovská 31, 37005 České Budějovice, Czech Republic; 2 Department of Molecular Biology, Faculty of Science, University of South Bohemia, Branišovská 31, 37005 České Budějovice, Czech Republic; 3 J. Craig Venter Institute, 10355 Science Center Drive, San Diego, CA 92121 USA; 4 Scripps Institution of Oceanography, 9500 Gilman Drive, La Jolla, CA 92093 USA

**Keywords:** Phylogenetics, Molecular evolution

## Abstract

Eukaryotic complex phototrophs exhibit a colorful evolutionary history. At least three independent endosymbiotic events accompanied by the gene transfer from the endosymbiont to host assembled a complex genomic mosaic. Resulting patchwork may give rise to unique metabolic capabilities; on the other hand, it can also blur the reconstruction of phylogenetic relationships. The ornithine–urea cycle (OUC) belongs to the cornerstone of the metabolism of metazoans and, as found recently, also photosynthetic stramenopiles. We have analyzed the distribution and phylogenetic positions of genes encoding enzymes of the urea synthesis pathway in eukaryotes. We show here that metazoan and stramenopile OUC enzymes share common origins and that enzymes of the OUC found in primary algae (including plants) display different origins. The impact of this fact on the evolution of stramenopiles is discussed here.

## Introduction

Ornithine–urea cycle (OUC) biochemistry and metabolism had been for a long time known to function exclusively in animals. Recently the OUC was also found in phototrophic stramenopiles and haptophytes. However, the function of this pathway documented in diatoms is probably substantially different from the one found in animals. While metazoans use the OUC to get rid of the excessive nitrogen, diatoms (likely all stramenopiles) utilize this pathway more likely for optimized nitrogen management in response to widely fluctuating nitrogen availability in the ocean environment^1,2^. We have shown recently that RNAi knockdown of carbamoylphosphate synthase (CPS), the key enzyme supplying the OUC pathway with carbon and nitrogen substrate, in the diatom *Phaeodactylum tricornutum* leads to a metabolic imbalance within the cell. Surprisingly, disruption of OUC cycle affected not only a production of OUC related compounds such as arginine, asparagine, aspartic acid, ornithine, urea, proline, and glutamine, but also fumaric acid, malic acid, citric acid, succinic acid, 2-oxoglutarate and glucose, products associated to the TCA cycle^1^. It is thus possible that OUC optimizes the metabolic response to the environmental cues and plays a crucial role in the evolutionary success of diatoms and possibly also other stramenopiles and haptophytes^2^.

**Figure 1:**
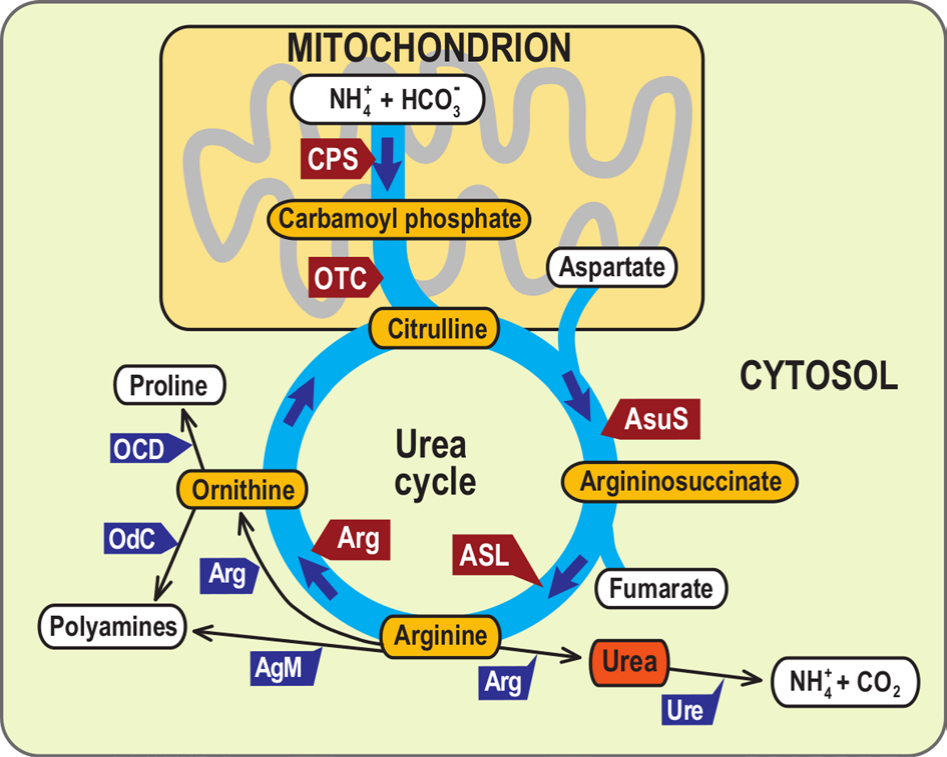
Schematics of canonical urea pathway, including cell compartmentalization. Red arrows denote key enzymes of the pathway, which are subject of this study, while blue arrows list enzymes downstream of OUC. Yellow ellipses show key metabolites of the cycle, white (and red) ellipses list upstream and downstream metabolites. *CPS* carbamoyl phosphate synthetase, *OTC* ornithine transcarbamylase, *ASuS* argininosuccinate synthase, *ASL* argininosuccinate lyase, *ARG* arginase, *AgM* agmatinase, *Ure* urease, *OdC* ornithine decarboxylase, *OCD* ornithin cyclodeaminase.

The OUC cycle is composed of five enzymes. In the metazoan OUC, carbamoylphosphate synthase (CPS), localized within the mitochondrion, produces carbamoylphosphate. It is converted by also mitochondrially located ornithine transcarbamylase (OTC) to l-citrulline. Citrulline is then exported to the cytosol, where it is used together with aspartate to form argininosuccinate in a reaction catalyzed by argininosuccinate synthase (ASuS). In the next step, arginine is synthesized by argininosuccinate lyase (ASL) and then metabolized by arginase (ARG) to urea or ornithine or by agmatinase to spermidine (Fig. 1). However, although metazoans use the OUC as a catabolic pathway (i.e., to metabolize the conversion of toxic ammonia to harmless urea), in stramenopiles, it seems to have rather an anabolic character instead. It plays a vital role in the overall cellular balance of carbon and nitrogen^1,2^.

Allen et al.^1^ also showed that the key enzyme of the OUC, carbamoyl phosphate synthase (CPS), has likely evolved by a duplication of an ancient CPS involved in pyrimidine biosynthesis and switch of its substrate specificity from glutamate to ammonium. This duplication was probably followed by a second duplication event of the ammonium-dependent CPS and reverse switch of the substrate specificity back to glutamate. This was followed by the gradual loss of the original CPS involved in pyrimidine biosynthesis in several groups, including stramenopiles, but also apicomplexan parasites (causative agents of malaria, toxoplasmosis, etc.) or other algae harboring complex plastids. As a result, animals use the original CPS to synthesize pyrimidines and the supposed first CPS duplicate to feed the urea cycle, while stramenopiles use its copy to run the OUC and the duplicate of this copy to synthesize pyrimidines. Interestingly, apicomplexans use the CPS involved in OUC in animals to produce pyrimidines and lost the original OUC during the evolution. Although this evolutionary scenario is relatively complex, it shows that CPS involved in OUC in animals share common origins with both CPSs homologous enzymes from stramenopiles^1^.

The most recent view on the phylogeny of eukaryotes suggests a common ancestor for the SAR clade (eukaryotic supergroup, comprised of **S**tramenopila, **A**lveolates, and **R**hizarians), haptophytes (together with some minor lineages) and archaeplastids^3,4^. However, such a proposal is obviously in contrast to the origin of CPS in stramenopiles, chlorarachniophytes, and apicomplexan parasites (Allen et al.^1^), because the SAR lineages branch with metazoans with the exclusion of the archaeplastids. To investigate this conflict to the necessary detail, we more precisely examined the origin of OUC and performed phylogenetic analyses of all enzymes involved in OUC (i.e., CPS, OTC, ASuS, ASL and Arg).

## Methods

We constructed a custom database covering all prokaryotic and eukaryotic domains of life. Protein sequences of taxa of interest were extracted from the GenBank nr database using a perl script filterdb (PhyloGenie software package^5^) or downloaded from the JGI database (https://genome.jgi-psf.org/) as well as the MMETSP project^6^. Within every taxon, redundant sequences (95% similarity threshold) were filtered out using the ‘cluster fast’ tool implemented in Usearch^7^. For each gene of the OUC (i.e., CPS, OTC, ASuS, ASL and ARG), we created a query file (Supplementary Table S1) containing homologs from primary and complex algae (algae with secondary and higher-order plastids), performed a homolog-search against our database using standalone NCBI Blast + under the default settings except for e-value threshold set to 1e−10 . The Blast results for individual genes were then parsed into the datasets that were subsequently aligned using MAFFT (version 7) and the local-pair algorithm^8^. Alignments were edited, and ambiguous parts were manually removed in Seaview 4^9^.

Maximum likelihood (ML) phylogenies were performed using IQTree 1.5^10^ under the best-fitting model chosen from following matrices: LG, LG4X, LG4M, C20, C40, LG + C20, and LG + C40, using the implemented model-finder. Non-parametric bootstrap support was estimated using the ultra-fast method from 10,000 replicates under the conditions described above. Alternatively, we performed Bayesian Inference (BI) using the Phylobayes 4.1^11^, with the CAT admixture model with the number of mixture categories set to 40 and exchange rates as defined by the LG empirical matrix (LG + C40 model). Four different chains were run until they converged (i.e., the maximal observed discrepancy was lower than 0.02), and the effective sample size of model parameters reached 100. Posterior probabilities represent statistical branching support.

## Results

**Figure 2:**
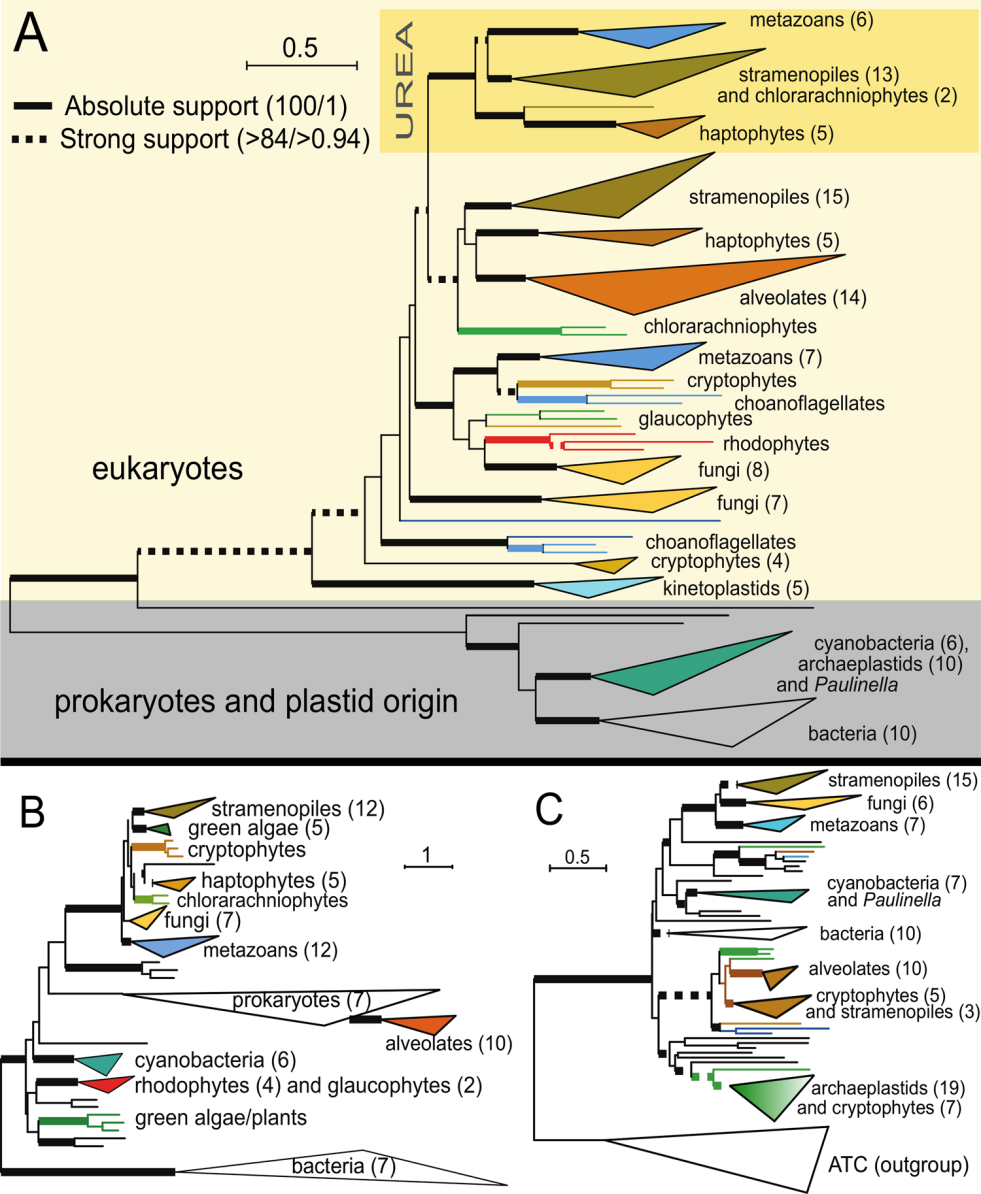
Schematized maximum likelihood phylogeny of carbamoyl phosphate synthetase (CPS) (**A**), argininosuccinate lyase (ASL) (**B**), and ornithine transcarbamylase (OTC) (**C**), inferred using the LG + C40 model as implemented in IQTree. Taxonomically homogenous clades are collapsed for better readability of the tree. The numbers in brackets correspond to the number of taxa in the collapsed clade. Eukaryotic taxa are highlighted in color, while non-cyanobacterial prokaryotic taxa are indicated by black branches. Ultrafast non-parametric bootstrap support was estimated from 10,000 replicates in IQTree under the above-specified model. The Bayesian posterior probabilities were computed in Phylobayes 4.1 (details described in “Methods”). Only absolute (bold lines) and high support (dotted lines) are shown. For more details on branching support, see the corresponding Supplemental Figures. (**A**) The CPS tree is based on the alignment of 1283 amino acids from 171 taxa. The clade of amino-ependent CPS taking part in OUC is highlighted in a yellow rectangle. The full version of the tree is shown at Supplementary Figure S1. Species list and sequence IDs are also listed in Suppl. Table S2. (**B**) The ASL tree is based on the alignment of 436 amino acids from 94 taxa. The full version of the tree is shown at Suppl. Figure S4. Species list and sequence IDs are also listed in Suppl. Table S2. (**C**) The OTC tree is based on the alignment of 269 amino acids from 161 taxa. OTC sequences are rooted with the aspartate transcarbamoylase (ATC), which is an OTC paralog stemming from an ancient duplication event. The full version of the tree is shown at Suppl. Figure S2. Species list and sequence IDs are also listed in Suppl. Table S2.

Carbamoyl phosphate synthase (CPS) is the crucial enzyme of the pathway; however, it is not a part of the OU cycle but rather feeds it with an initial substrate, including carbon and nitrogen in the form of carbamoyl phosphate. While Allen et al. published CPS phylogeny in 2011, phylogenetic analyses of all OUC genes (including CPS) were performed on significantly expanded datasets here, enriched for dozens of (mostly) complex algal species, such as chlorarachniophytes, dinoflagellates, and haptophytes. This extended sampling of previously underrepresented or missing important lineages strengthens the analyses and expands the scope of our results. Compared to Allen et al.^1^, the composition of significant clades as well as monophyly of the ammonia-dependent ‘urea’ clade, comprising of stramenopiles, haptophytes, chlorarachniophytes, and metazoans, remain well supported and consistent (schematized on Fig. 2a). The closest representatives of primary endosymbiotic phototrophs are rhodophytes and glaucophytes branching with fungi, but they possess only the eukaryotic pyrimidine-synthesis associated CPS. Green algae and plants possess a single cyanobacterium-derived copy of CPS placed in the bacterial clade (Fig. 2a).

**Figure 3:**
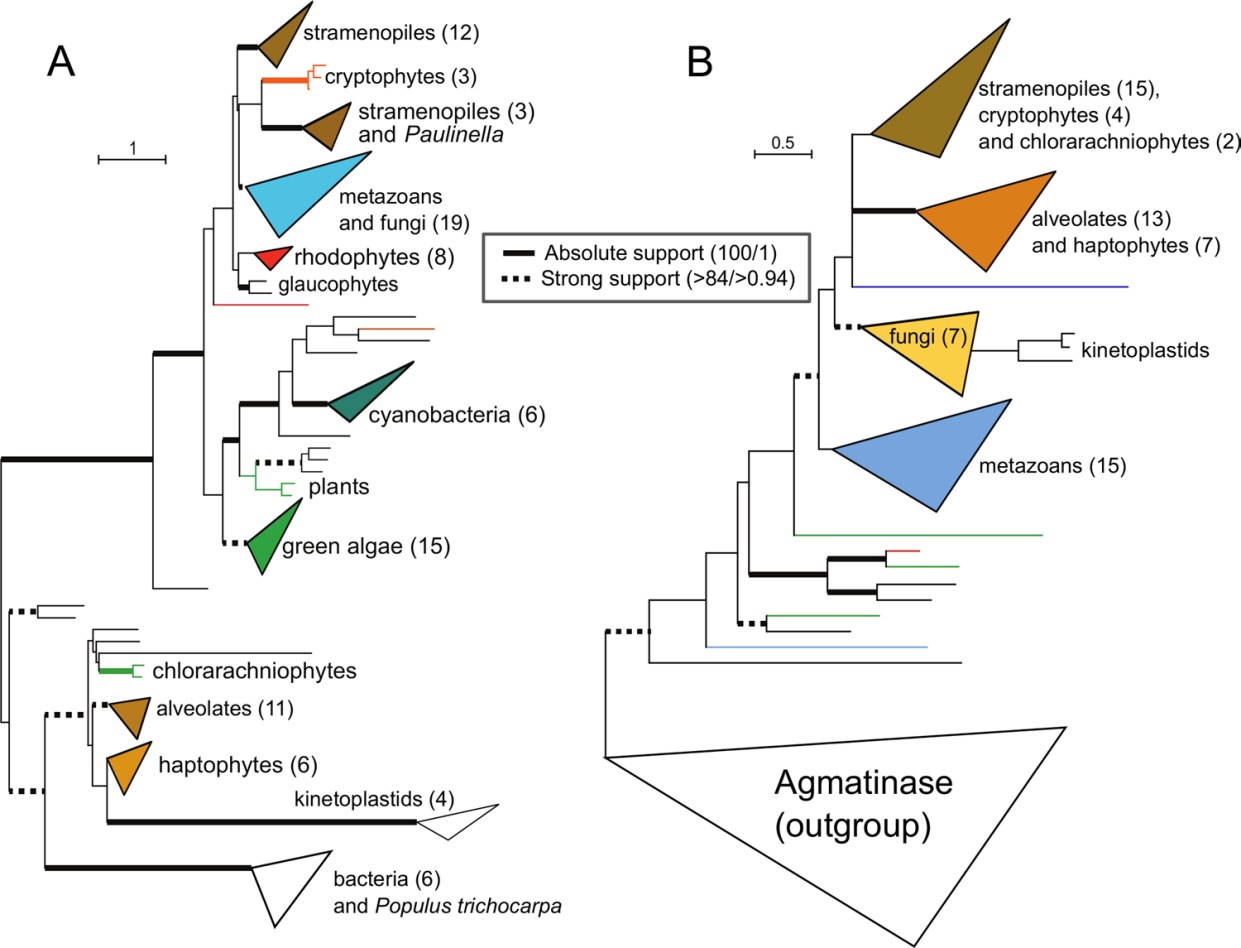
Schematized maximum likelihood phylogeny of Argininosuccinate Synthase (AsuS) (**A**), and Arginase (Arg) (**B**), inferred using the LG + C40 model as implemented in IQTree. Taxonomically homogenous clades are collapsed for better readability of the tree. The numbers in brackets correspond to the number of taxa in the collapsed clade. Eukaryotic taxa are highlighted in color, while non-cyanobacterial prokaryotic taxa are indicated by black branches. Ultrafast non-parametric bootstrap support was estimated from 10,000 replicates in IQTree under the above-specified model. The Bayesian posterior probabilities were computed in Phylobayes 4.1 (details described in “Methods”). Only absolute (bold lines) and high supported (dotted lines) are shown. For more details on branching support, see the corresponding Supplemental Figures. (**A**) The AsuS tree is based on the alignment of 374 amino acids from 118 taxa. The full version of the tree is shown at Suppl. Figure S3. Species list and sequence IDs are also listed in Suppl. Table S2. (**B**) The Arg tree is based on the alignment of 257 amino acids from 114 taxa. Arginase (ARG) sequences are rooted with the agmatinase, which is an ARG paralog stemming from an ancient duplication event. The full version of the tree is shown at Suppl. Figure S5. Species list and sequence IDs are also listed in Suppl. Table S2.

Except for the ASL (Fig. 2b), phylogenies of the remaining enzymes of the pathway follow the stramenopile-metazoan/opisthokont grouping, although the support varies from 100 (OTC, Fig. 2c) to 73 in ASuS (Fig. 3a).

The topology of the ornithine transcarbamylase (OTC), the second step of the OUC (Fig. 2c), is rooted using its paralogue—aspartate transcarbamoylase (ATC), which plays a role in the synthesis of pyrimidines. Interestingly, the stramenopiles—opisthokonts (fungi and animals) relationship is present in both OTC and ATC (details in Supplementary Fig. S2), suggesting that the branching pattern was established prior to the ancient duplication, which gave rise to both enzymes. In OTC, green algae and rhodophytes, together with haptophytes branch with bacterial sequences. Some stramenopiles (pelagomonads) as well as cryptophytes, chlorarachniophytes, and dinoflagellates form a sister group to excavates *Giardia lamblia* and *Trichomonas vaginalis*. This group is not placed within the main stramenopile clade.

The enzyme coding for the next reaction of the OUC, argininosuccinate synthase (ASuS), follows the same scenario, with stramenopiles accompanied by cryptophytes, branching with opisthokonts (Fig. 3a). Primary endosymbiotic phototrophs are split between green algae/plants branching with bacteria, while rhodophytes and glaucophytes form a common clade among eukaryotes. The rest of complex algae, including alveolates, chlorarachniophytes, and haptophytes, accompanied by kinetoplastids and a sequence of the flowering plant *Populus trichocarpa* branch among the non-photosynthetic prokaryotes.

A similar pattern can also be followed in the case of arginase, which encodes the last step of the OUC. Arginase phylogeny (ARG, Fig. 3b) is rooted with agmatinase (AgM), a paralogue of arginase involved in the metabolism of urea and amino groups. Here, all the representatives of the SAR clade are accompanied by haptophytes, cryptophytes, and dinoflagellates, and form a common clade with opisthokonts. Primary endosymbiotic phototrophs, represented by the non-flowering plant (moss) *Physcomitrella patens* only*,* branch outside the eukaryotic clade, with various bacteria. We could not identify any ARG homolog in rhodophytes and glaucophytes. Several other green algae are found in AGM clade, however.

The only enzyme of the pathway that does not support the stramenopile-opisthokont relationship is ASL (Fig. 2b). Here, two clades of primary symbiotic phototrophs are present. Higher plants, rhodophytes, and glaucophytes branch deep among bacteria. On the other hand, unicellular green algae form a well-supported clade with stramenopiles, sister to cryptophytes, haptophytes, and chlorarachniophytes. This huge assemblage of primary and complex algae then robustly branches with opisthokonts. The complexity of ASL evolution in photosynthetic eukaryotes is completed by the last major lineage of complex algae, myzozoans (belonging to alveolates), here represented by chromerids and dinoflagellates. They are found in a well-supported clade among various non-photosynthetic bacteria (Fig. 2b).

## Discussion

Even though the functional ornithine–urea cycle is described only from ureotelic metazoans and stramenopiles, most (if not all) steps of the pathway are present in representatives of all eukaryotic supergroups. From this perspective, the presence of two CPS copies of which one is mitochondrially targeted seems to be an essential condition of functional OUC (but see below for an alternative explanation). Our phylogenies suggest only some metazoans, stramenopiles, chlorarachniophytes, and haptophytes fulfill this condition. Outside of metazoans, the functionality of this paralogue has been experimentally proved only in the diatom *Phaeodactylum tricornutum*^1^. However, we found it in the transcriptomic MMETSP dataset in other photosynthetic stramenopiles (including multicellular kelps), but also in key ocean primary producers—haptophytes as well as in minor cercozoan photosynthetic lineage chlorarachniophytes. The OUC may thus be functional also in other algae with complex plastids to which it may provide the same metabolic advantage as to diatoms.

The same also applies to non-photosynthetic stramenopiles. As evident from the data at the web of JGI (https://genome.jgi.doe.gov), the ammonium-dependent mitochondrion-targeted CPS paralogue is also expressed in heterotrophic oomycetes *Phytophthora ramorum* and *P*. *sojae* as well as labyrinthulomycete *Thrausthochytrium limacinum*. It is missing in some heterotrophic stramenopiles, like human symbiont *Blastocystis hominis,* as well as in all alveolates screened so far. Still, the data presented here suggest the presence of the ammonium-dependent mitochondrion-targeted CPS paralogue in the common ancestor of Haptista and SAR clades^3^ and consecutive loss in several SAR lineages.

We have enriched the dataset with (mostly) previously unavailable (dinoflagellates and chromerids), or poorly represented primary and complex eukaryotic phototrophs (haptophytes, chlorarachniophytes, rhodophytes, cryptophytes, and chlorophytes) as well as heterotrophic apicomplexans and stramenopiles. However, the composition of the main clades, as well as their mutual position, remained the same suggesting the evolutionary explanation of the observed distribution of CPS proposed in Allen et al.^1^. Both chlorarachniophytes and haptophytes possess two copies of CPS, one of which in the novel ammonium-dependent mitochondrially-targeted clade comprising of complex algae. Surprisingly, cryptophytes also possess two copies of CPS. Both seem to lack the mitochondrial targeting and branch outside the Metazoa-SAR OUC clade. However, some cryptophyte sequences coming from the RNASeq data lack the N terminus, and mitochondrial targeting peptide may be missing. Although not probable, the presence of OUC in cryptophytes thus cannot be excluded. None of the cryptophyte CPS paralogs branch with archaeplastids, which fits some recent views on cryptophytes as an independent eukaryotic lineage^12–14^. However, this is not in agreement with the most recent phylogenomic studies of eukaryotes^3,4^, where cryptophytes branch from within the archaeplastids.

Archaeplastid lineages (green algae, glaucophytes, and rhodophytes) also lack the urea-synthesizing CPS paralogue. Contrary to green algae, which possess only plastid-derived CPS branching withcyanobacteria, rhodophytes and glaucophytes retained both plastid-derived and eukaryotic copies. Interestingly, the latter one is speculated to be involved in amino acid biosynthesis in the rhodophyte *Cyanidioschyzon merolae*^15^. Therefore, the functional OUC is likely missing in archaeplastids. Recently, a functionally equivalent yet distinct alternative to the OUC, the ornithine–ammonia cycle (OAC), has been described in cyanobacteria^16^, and authors speculate the novel pathway may also be active in plants.

Except for ASL, all the remaining steps of the pathway show a similar scenario as CPS. The stramenopiles, accompanied by haptophytes and chlorarachniophytes (i.e., the organisms possessing ammonia-dependent CPS and therefore possibly functional OUC), branch either as a sister group to-, or within the opisthokonts, with the exclusion of archaeplastid lineages, which appear elsewhere in the tree. The only exception to this pattern is ASL, with prasinophyte and trebouxiophyte lineages branching within the chromalveolate-opisthokont clade. However, given the fact that other archaeplastids, including higher plants, form a clade of apparently bacterial origin, we think this irregularity could be assigned either to the lateral gene transfer or some phylogenetic artifact. Alternatively, these prasinophyte/trebouxiophyte ASL homologs may represent the original archaeplastid host (exosymbiont), in other genes of OUC replaced by bacterial copies.

Contrary to general expectation, the donors are probably not of cyanobacterial origin as they branch with planctomycete *Rhodopirullela baltica*. Some of these non-canonical topologies are probably caused by a combination of short amino acid sequences and associated poor phylogenetic signal. However, we generally see only a few unambiguous cases of endosymbiotic gene transfer (EGT) or plastidic origin of respective steps of the OUC pathway among primary or complex algae (maybe with the exception of CPS) compared to, e.g., heme pathway^17^.

This observation is in agreement with canonical cytosolic/mitochondrial compartmentalization of the pathway^18^. It also partly corresponds to the situation in plants. Reyes-Prieto et al.^19^ showed that out of the 62 plastid-localized enzymes involved in the amino acid biosynthesis, two-thirds originate in non-cyanobacterial prokaryotes. Similarly, in chromerids (photosynthetic ancestors of apicomplexans), the vast majority of the synthesis of amino acids takes place in the cytosol^20^. The former study also includes two enzymes of OUC involved in the synthesis of arginine. While the CPS of higher plants is of cyanobacterial origin, OTC was acquired from other prokaryotes. It contrasts with the topology shown here (Fig. 2c), with green algae branching with other archaeplastids and cryptophytes. The discrepancy may be caused by different taxon sampling, as well as varying details of phylogenetic methodology. Arginine is the main precursor for the synthesis of nitric oxide as well as polyamines and thus plays an essential role in cell signaling and growth regulation^21–23^. Apart from plants, the nitric oxide was shown to be an essential cell signaling molecule also in diatoms^24–26^, rhodophytes^27^, streptophytes^28^, or haptophytes^26^. Therefore, given the above-suggested importance of arginine synthesis as well as the metabolic advantage of the OUC for some algal groups^1^, one would expect its evolutionary history to be more tightly associated with the evolution of plastids.

Despite the proposed presence of OUC only in stramenopiles and metazoans, most of the eukaryotic lineages possess genes for the majority, if not all, enzymes of the OUC pathway (Table 1). In cyanobacteria, an alternative to OUC, ornithine–ammonia cycle (OAC), was described^16^. Like eukaryotic phototrophs, cyanobacteria also have high demands for nitrogen when rapid division is needed. The recently discovered OAC starts with the synthesis of the carbamoyl phosphate and ends with the conversion of arginine to ornithine and ammonia. Apart from the arginine dihydrolase, which replaces the arginase, all the remaining enzymatic steps are shared with the OUC. The OAC allows cyanobacteria rapid access to stored nitrogen reserves as well as efficient nitrogen assimilation and storage when it becomes available^16^. Although the functional OAC was so-far confirmed only in cyanobacteria, its possible existence also in eukaryotes is speculated^16^. If confirmed, it would explain the presence of several above-mentioned OUC genes in lineages without functional OUC, such as plants.

**Table 1:**
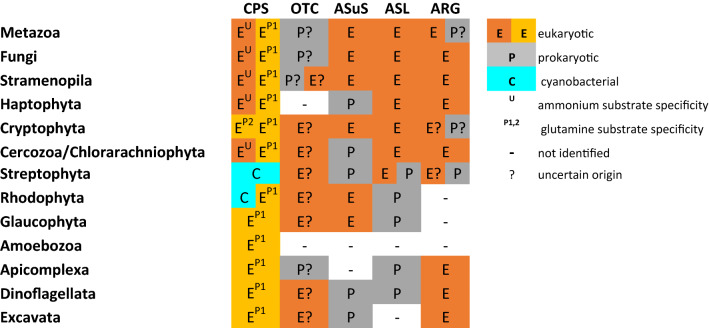
Distribution of OUC enzymes among the major eukaryotic lineages.

Eukaryotic, cyanobacterial, and “other prokaryotes” origin is color-coded. Not identified/missing homologs are shown by the dash symbol. Cases of weak supported or ambiguous phylogenetic signal are denoted by a question mark.

Evolutionary affiliation of SAR and haptophytes clades to the Archaeplastida^3,4,12,29^ is mainly built on the results of phylogenomics. However, EGT from endosymbiont has substantially modified the composition of the nuclear genome of complex organisms, such as algae harboring complex plastids^30,31^. Besides cyanobacterial genes, also many eukaryotic “plant-like” genes have been transferred from an engulfed algal symbiont to the complex host (exosymbiont) nucleus during the process of complex endosymbioses^14,32–35^. Such a complex composition of exosymbiont nucleus, which contains eukaryotic genes of both symbiotic partners, makes phylogenomic analysis extremely difficult to interpret. When the archaeplastid-like sequences are filtered out of the datasets, we may lose crucial phylogenetic information. When they are retained, the endosymbiotic gene transfer may deform phylogenetic signal, and the affiliation of the SAR group to archaeplastids can be an obvious artifact. Such errors can be multiplied using automatic phylogenetic pipelines, usually by incorrect taxon sampling of datasets that were not manually checked. Therefore, to look at the origins of organisms with highly complex evolutionary history, we suggest a precise phylogenetic investigation of particular metabolic pathways as, in our opinion, a viable alternative. Of course, we do not mean to propose a different evolutionary history of one of the main eukaryotic clades based on the analysis of a single and rather simple metabolic pathway. Also, we are fully aware that alternative explanations of observed state, such as the transfer of the whole pathway from an ancestor of opisthokonts to stramenopiles (or vice versa), convergent evolution of involved enzymes as well as a sudden redundancy (caused either by the duplication or lateral/endosymbiotic gene transfer) followed by a differential loss of some paralogs or pseudoparalogs^36^ exist. We cannot exclude the possibility any of the above mentioned “extreme” scenarios took place.

Moreover, we feel there is no reason to assume all the genes of the OUC pathway share the common evolutionary history. Some of the processes mentioned above combined to create a complex evolutionary mosaic. However, we feel the impact of EGT on phylogenomic studies has not been critically evaluated to the necessary depth, especially in the case of complex-plastid-bearing eukaryotes.

## Supplementary information

Supplementary Information

## References

[1] Allen AE (2011). Evolution and metabolic significance of the urea cycle in photosynthetic diatoms. Nature.

[2] Smith SR (2019). Evolution and regulation of nitrogen flux through compartmentalized metabolic networks in a marine diatom. Nat. Commun..

[3] Burki F (2016). Untangling the early diversification of eukaryotes: A phylogenomic study of the evolutionary origins of Centrohelida, Haptophyta and Cryptista. Proc. Biol. Sci..

[4] Strassert JFH, Jamy M, Mylnikov AP, Tikhonenkov DV, Burki F (2019). New phylogenomic analysis of the enigmatic phylum telonemia further resolves the eukaryote tree of life. Mol. Biol. Evol..

[5] Frickey T, Lupas AN (2004). PhyloGenie: Automated phylome generation and analysis. Nucleic Acids Res..

[6] Keeling PJ (2014). The marine microbial eukaryote transcriptome sequencing project (MMETSP): Illuminating the functional diversity of eukaryotic life in the oceans through transcriptome sequencing. PLoS Biol..

[7] Edgar RRC (2010). Search and clustering orders of magnitude faster than BLAST. Bioinformatics.

[8] Katoh K, Standley DM (2013). MAFFT Multiple Sequence Alignment Software Version 7: Improvements in performance and usabiltiy. Mol. Biol. Evol..

[9] Gouy M, Guindon S, Gascuel O (2010). SeaView version 4: A multiplatform graphical user interface for sequence alignment and phylogenetic tree building. Mol. Biol. Evol..

[10] Nguyen LT, Schmidt HA, Von Haeseler A, Minh BQ (2015). IQ-TREE: A fast and effective stochastic algorithm for estimating maximum-likelihood phylogenies. Mol. Biol. Evol..

[11] Lartillot N, Lepage T, Blanquart S (2009). PhyloBayes 3: A Bayesian software package for phylogenetic reconstruction and molecular dating. Bioinformatics.

[12] Burki F, Okamoto N, Pombert J-FJ-F, Keeling PJ (2012). The evolutionary history of haptophytes and cryptophytes: Phylogenomic evidence for separate origins. Proc. R. Soc. B Biol. Sci..

[13] Burki F, Shalchian-Tabrizi K, Pawlowski J (2008). Phylogenomics reveals a new ‘megagroup’ including most photosynthetic eukaryotes. Biol. Lett..

[14] Curtis BA (2012). Algal genomes reveal evolutionary mosaicism and the fate of nucleomorphs. Nature.

[15] Nozaki H (2005). Phylogenetic implications of the CAD complex from the primitive red alga Cyanidioschyzon merolae (Cyanidiales, Rhodophyta). J. Phycol..

[16] Zhang H (2018). The cyanobacterial ornithine–ammonia cycle involves an arginine dihydrolase. Nat. Chem. Biol..

[17] Cihlář J, Füssy Z, Horák A, Oborník M (2016). Evolution of the tetrapyrrole biosynthetic pathway in secondary algae: Conservation, Redundancy and Replacement. PLoS One.

[18] Davis PK, Wu G (1998). Compartmentation and kinetics of urea cycle enzymes in porcine enterocytes. Comp. Biochem. Physiol. B. Biochem. Mol. Biol..

[19] Reyes-Prieto A, Moustafa A (2012). Plastid-localized amino acid biosynthetic pathways of Plantae are predominantly composed of non-cyanobacterial enzymes. Sci. Rep..

[20] Füssy Z, Faitová T, Oborník M (2019). Subcellular compartments interplay for carbon and nitrogen allocation in chromera velia and vitrella brassicaformis. Genome Biol. Evol..

[21] Domingos P, Prado AM, Wong A, Gehring C, Feijo JA (2015). Nitric oxide: A multitasked signaling gas in plants. Mol. Plant.

[22] Neill S (2008). Nitric oxide, stomatal closure, and abiotic stress. J. Exp. Bot..

[23] Gupta KJ (2011). The emerging roles of nitric oxide (NO) in plant mitochondria. Plant Sci..

[24] Vardi A (2006). A stress surveillance system based on calcium and nitric oxide in marine diatoms. PLoS Biol..

[25] Vardi A (2008). Cell signaling in marine diatoms. Commun. Integr. Biol..

[26] Kumar A, Castellano I, Patti FP, Palumbo A, Buia MC (2015). Nitric oxide in marine photosynthetic organisms. Nitric Oxide.

[27] Chow F, Pedersén M, Oliveira MC (2013). Modulation of nitrate reductase activity by photosynthetic electron transport chain and nitric oxide balance in the red macroalga *Gracilaria chilensis* (Gracilariales, Rhodophyta). J. Appl. Phycol..

[28] Estevez MS, Puntarulo S (2005). Nitric oxide generation upon growth of Antarctic Chlorella sp. cells. Physiol. Plant..

[29] Brown MW (2018). Phylogenomics places orphan protistan lineages in a novel eukaryotic super-group. Genome Biol. Evol..

[30] Timmis JN, Ayliffe MA, Huang CY, Martin W (2004). Endosymbiotic gene transfer: Organelle genomes forge eukaryotic chromosomes. Nat. Rev. Genet..

[31] Moreira D, Deschamps P (2014). What was the real contribution of endosymbionts to the eukaryotic nucleus? Insights from photosynthetic eukaryotes. Cold Spring Harb. Perspect. Biol..

[32] Moustafa A (2009). Genomic footprints of a cryptic plastid endosymbiosis in diatoms. Science (80).

[33] Deschamps P, Moreira D (2012). Reevaluating the green contribution to diatom genomes. Genome Biol. Evol..

[34] Dorrell RG, Smith AG (2011). Do red and green make brown?: Perspectives on plastid acquisitions within chromalveolates. Eukaryot. Cell.

[35] Dorrell RG (2017). Chimeric origins of ochrophytes and haptophytes revealed through an ancient plastid proteome. Elife.

[36] Gile GH (2009). Distribution and phylogeny of EFL and EF-1α in euglenozoa suggest ancestral co-occurrence followed by differential loss. PLoS One.

