# Supplementary material for: Common origin of ornithine–urea cycle in opisthokonts and stramenopiles
Source: Sci Rep. 2020 Oct 7;10:16687. doi: 10.1038/s41598-020-73715-8 (PMC7542463; doi:10.1038/s41598-020-73715-8)
Supplement: Supplementary file 1 — Supplementary Information [file 41598_2020_73715_MOESM1_ESM.pdf]

Supplemental information

**Common origin of Ornithine-Urea Cycle in opisthokonts and stramenopiles.**

Aleš Horák[1,2], Andrew Allen[3,4], Miroslav Oborník[1,2]

[1] Biology Centre, Czech Academy of Sciences, Institute of Parasitology, Branišovská 31, 37005 České Budějovice, Czech Republic

[2] University of South Bohemia, Faculty of Science, Department of Molecular Biology, Branišovská 31, 37005 České Budějovice, Czech Republic

[3]J. Craig Venter Institute, San Diego, 10355 Science Center Drive, California 92121, USA

[4]Scripps Institution of Oceanography, 9500 Gilman Drive, La Jolla, California 92093, USA

Address for correspondence: oborník@paru.cas.cz

**Supplemental Figure legends:**

Supplemental Figure S1. Unsimplified maximum likelihood phylogeny of Carbamoyl Phosphate Synthetase (CPS) based on the alignment of 1283 aminoacids from 171 taxa. The tree was inferred using the LG+C40 model in IQTree 1.5. Numbers at nodes represent ultrafast bootstrap support estimated from 10000 replication in IQTree under the above-specified model as well as the Bayesian Posterior Probabilities computed in Phylobayes 4.1 (details described in Methods). Only bootstrap support over 84/posterior probabilities of 0.95 and higher are shown. Dash symbol indicates the topology in one of the methods used did not meet the support values described above, d.t. stands for different topology.

Supplemental Figure S2. Unsimplified maximum likelihood phylogeny of Argininosuccinate Lyase (ASL) based on the alignment of 436 aminoacids from 94 taxa. The tree was inferred using the LG+C40 model in IQTree 1.5. Numbers at nodes represent ultrafast bootstrap support estimated from 10000 replication in IQTree under the above-specified model as well as the Bayesian Posterior probabilities computed in Phylobayes 4.1 (details described in Methods). Only bootstrap support over 84/posterior probabilities of 0.95 and higher are shown. Dash symbol indicates the topology in one of the methods used did not meet the support values described above, d.t. stands for different topology.

Supplemental Figure S3. Unsimplified maximum likelihood phylogeny of Ornithine Transcarbamoylase (OTC) based on the alignment of 269 aminoacids from 161 taxa. The tree was inferred using the LG+C40 model in IQTree 1.5. Numbers at nodes represent ultrafast bootstrap support estimated from 10000 replication in IQTree under the above-specified model as well as the Bayesian Posterior probabilities computed in Phylobayes 4.1 (details described in Methods). OTC sequences are rooted with the aspartate transcarbamoylase

(ATC), which is an OTC paralog stemming from an ancient duplication event. Only bootstrap support over 84/posterior probabilities of 0.95 and higher are shown. Dash symbol indicates the topology in one of the methods used did not meet the support values described above, d.t. stands for different topology.

Supplemental Figure S4. Unsimplified maximum likelihood phylogeny of Argininosuccinate Synthase (ASuS) based on the alignment of 374 aminoacids from 118 taxa. The tree was inferred using the LG+C40 model in IQTree 1.5. Numbers at nodes represent ultrafast bootstrap support estimated from 10000 replication in IQTree under the above-specified model as well as the Bayesian Posterior probabilities computed in Phylobayes 4.1 (details described in Methods). Only bootstrap support over 84/posterior probabilities of 0.95 and higher are shown. Dash symbol indicates the topology in one of the methods used did not meet the support values described above, d.t. stands for different topology.

Supplemental Figure S5. Unsimplified maximum likelihood phylogeny of Arginase (ARG) based on the alignment of 257 aminoacids from 114 taxa. The tree was inferred using the LG4X model in IQTree 1.5. Numbers at nodes represent ultrafast bootstrap support estimated from 10000 replication in IQTree under the above-specified model as well as the Bayesian Posterior probabilities computed in Phylobayes 4.1 (details described in Methods). ARG sequences are rooted with the agmatinase (AGM), which is an ARG paralog stemming from an ancient duplication event. Only bootstrap support over 84/posterior probabilities of 0.95 and higher are shown. Dash symbol indicates the topology in one of the methods used did not meet the support values described above, d.t. stands for different topology.

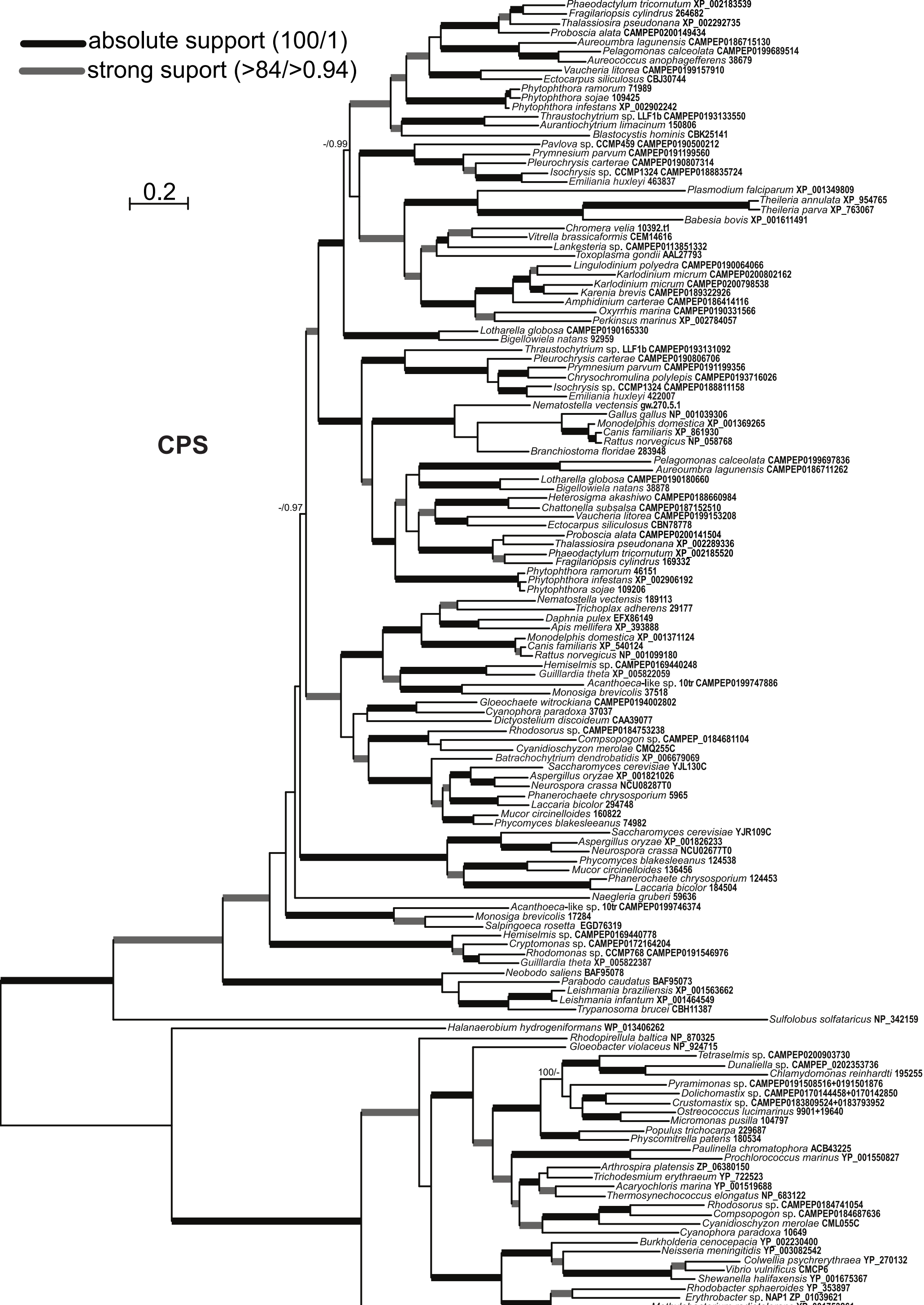


**Supplemental Figure S1**

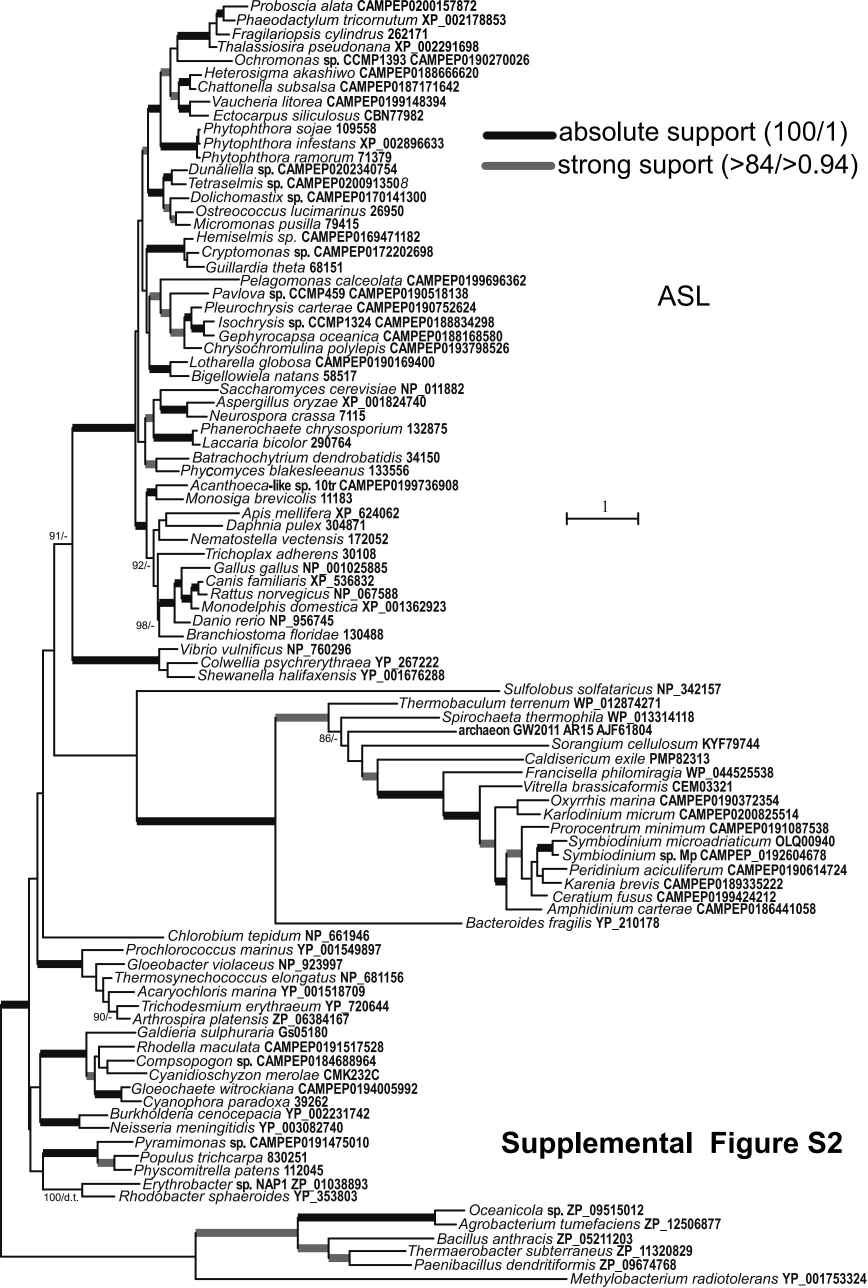


**Supplemental Figure S2**

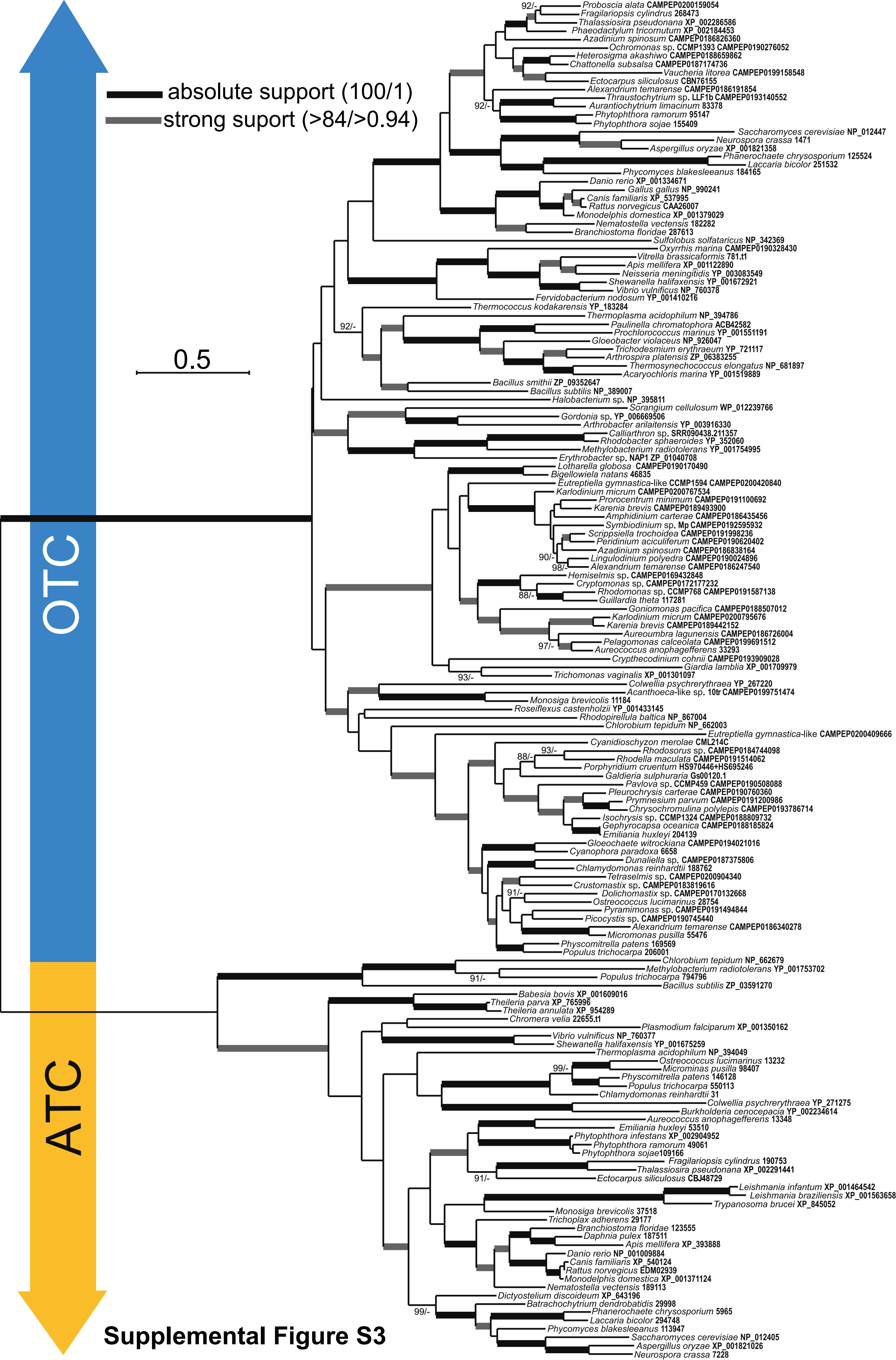


**Supplemental Figure S3**

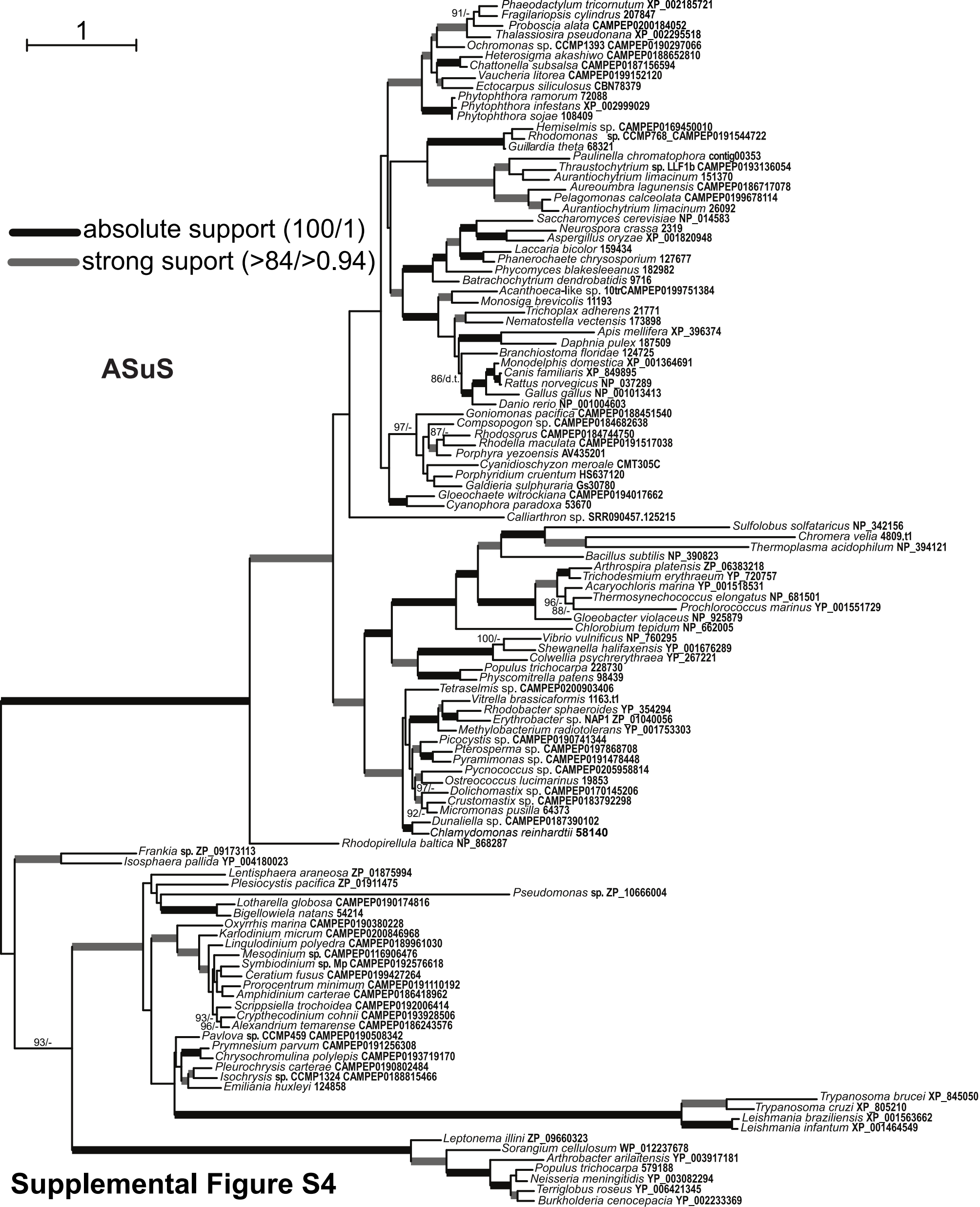


**Supplemental Figure S4**

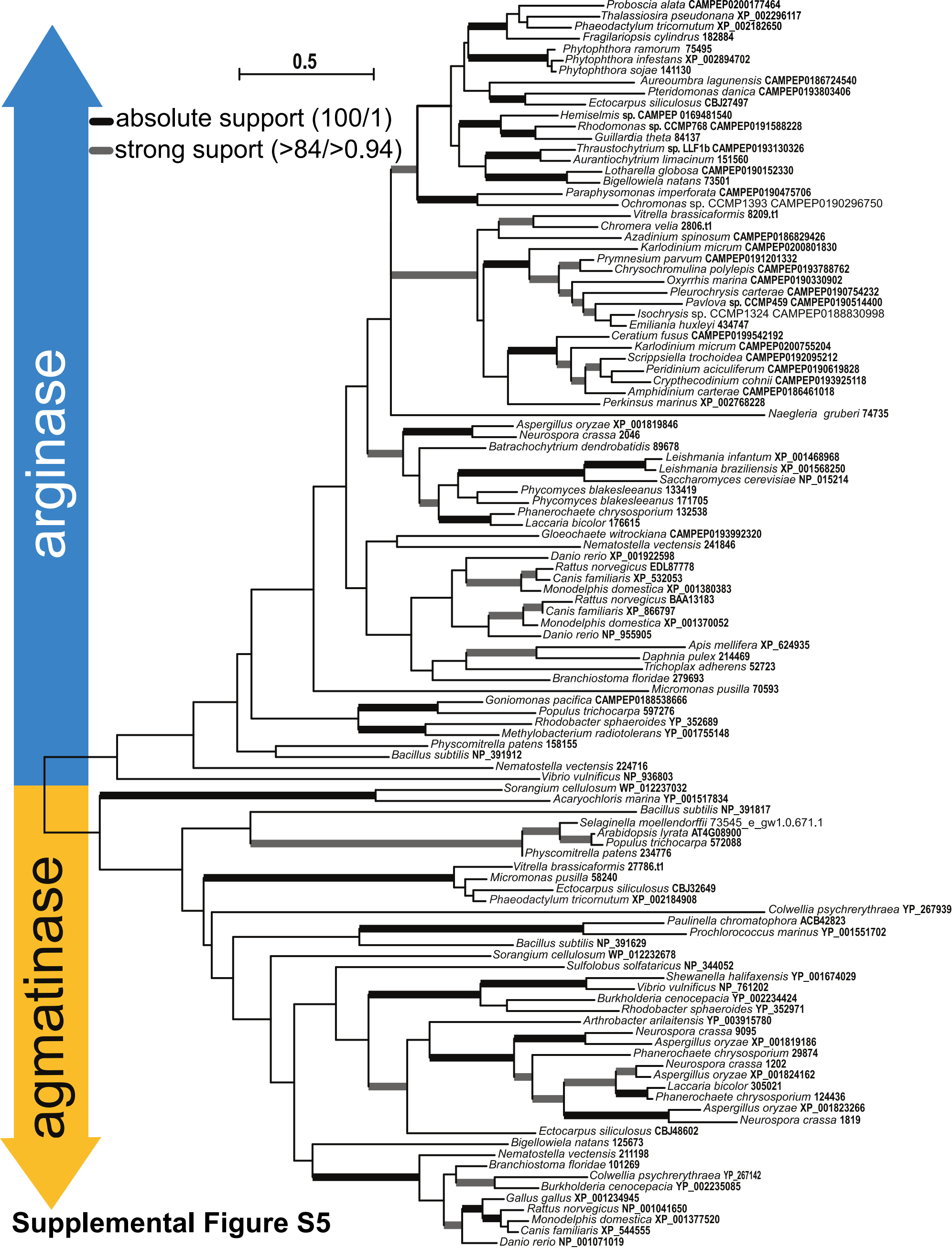


**Supplemental Figure S5**

**Supplemental Table S1. Accension numbers of sequences used as query for Blast search**

| | *Phaeodactylum tricornutum* | *Populus trichocarpa** | |
|---|---|---|---|
| **CPS** | XP_002183539 | 229687 | |
| **OTC** | XP_002184453 | 206001 | |
| **ASuS** | XP_002185721 | 228730 | |
| **ASL** | XP_002178853 | 830251 | * JGI genome portal database |
| **ARG** | XP_002184453 | 597276 | |

**Supplemental Table S2.**

List of taxa including sequence ID’s used in phylogenetic analyses of OUC genes (Figures 2-6).

| CPS | ASL | OCT | ASuS | ARG |
|---|---|---|---|---|
| Acanthoeca-like sp. 10tr CAMPEP0199746374 | Acanthoeca-like sp. 10tr CAMPEP0199736908 | Acanthoeca-like sp. 10tr CAMPEP0199751474 | Acanthoeca-like sp. 10tr CAMPEP0199751384 | Acaryochloris marina YP_001517834 |
| Acanthoeca-like sp. 10tr CAMPEP0199747886 | Acaryochloris marina YP_001518709 | Acaryochloris marina YP_001519889 | Acaryochloris marina YP_001518531 | Amphidinium carterae CAMPEP0186461018 |
| Acaryochloris marina YP_001519688 | Agrobacterium tumefaciens ZP_12506877 | Alexandrium temarense CAMPEP0186191854 | Alexandrium temarense CAMPEP0186243576 | Apis mellifera XP_624935 |
| Amphidinium carterae CAMPEP0186414116 | Amphidinium carterae CAMPEP0186441058 | Alexandrium temarense CAMPEP0186247540 | Amphidinium carterae CAMPEP0186418962 | Arabidopsis lyrata AT4G08900 |
| Apis mellifera XP_393888 | Apis mellifera XP_624062 | Alexandrium temarense CAMPEP0186340278 | Apis mellifera XP_396374 | Arthrobacter arilaitensis YP_003915780 |
| Arthrobacter arilaitensis YP_003916898 | archaeon GW2011 AR15 AJF61804 | Amphidinium carterae CAMPEP0186435456 | Arthrobacter arilaitensis YP_003917181 | Aspergillus oryzae XP_001819186 |
| Arthrospira platensis ZP_06380150 | Arthrospira platensis ZP_06384167 | Apis mellifera XP_001122890 | Arthrospira platensis ZP_06383218 | Aspergillus oryzae XP_001823266 |
| Aspergillus oryzae XP_001821026 | Aspergillus oryzae XP_001824740 | Apis mellifera XP_393888 | Aurantiochytrium limacinum 26092 | Aspergillus oryzae XP_001824162 |
| Aspergillus oryzae XP_001826233 | Bacillus anthracis ZP_05211203 | Arthrobacter arilaitensis YP_003916330 | Aureococcus anophagefferens 151370 | Aspergillus_oryzae XP_001819846 |
| Aurantiochytrium limacinum 150806 | Bacteroides fragilis YP_210178 | Arthrospira platensis ZP_06383255 | Aureoumbra lagunensis CAMPEP0186717078 | Aurantiochytrium limacinum 151560 |
| Aureococcus anophagefferens 38679 | Batrachochytrium dendrobatidis 34150 | Aspergillus oryzae XP_001821026 | Bacillus subtilis NP_390823 | Aureoumbra lagunensis CAMPEP0186724540 |
| Aureoumbra lagunensis CAMPEP0186711262 | Bigellowiela natans 58517 | Aspergillus oryzae XP_001821358 | Batrachochytrium dendrobatidis 9716 | Azadinium spinosum CAMPEP0186829426 |
| Aureoumbra lagunensis CAMPEP0186715130 | Branchiostoma floridae 130488 | Aurantiochytrium limacinum 83378 | Bigellowiela natans 54214 | Bacillus subtilis NP_391629 |
| Babesia bovis XP_001611491 | Burkholderia cenocepacia YP_002231742 | Aureococcus anophagefferens 13348 | Branchiostoma floridae 124725 | Bacillus subtilis NP_391817 |
| Batrachochytrium dendrobatidis XP_006679069 | Caldisericum exile PMP82313 | Aureococcus anophagefferens 33293 | Burkholderia cenocepacia YP_002233369 | Bacillus subtilis NP_391912 |
| Bigellowiela natans 38878 | Canis familiaris XP_536832 | Aureoumbra lagunensis CAMPEP0186726004 | Calliarthron sp. SRR090457.125215 | Batrachochytrium dendrobatidis 89678 |
| Bigellowiela natans 92959 | Ceratium fusus CAMPEP0199424212 | Azadinium spinosum CAMPEP0186826360 | Canis familiaris XP_849895 | Bigellowiela natans 125673 |
| Blastocystis hominis CBK25141 | Colwellia psychrerythraea YP_267222 | Azadinium spinosum CAMPEP0186838164 | Ceratium fusus CAMPEP0199427264 | Bigellowiela natans 73501 |
| Bradyrhizobium japonicum NP_774017 | Compsopogon sp. CAMPEP0184688964 | Babesia bovis XP_001609016 | Colwellia psychrerythraea YP_267221 | Branchiostoma floridae 101269 |
| Branchiostoma floridae 283948 | Cryptomonas sp. CAMPEP0172202698 | Bacillus smithii ZP_09352647 | Compsopogon sp. CAMPEP0184682638 | Branchiostoma floridae 279693 |
| Burkholderia cenocepacia YP_002230400 | Cyanidioschyzon merolae CMK232C | Bacillus subtilis NP_389007 | Crustomastix sp. CAMPEP0183792298 | Burkholderia cenocepacia YP_002234424 |
| Canis familiaris XP_540124 | Cyanophora paradoxa 39262 | Bacillus subtilis ZP_03591270 | Crypthecodinium cohnii CAMPEP0193928506 | Burkholderia cenocepacia YP_002235085 |
| Canis familiaris XP_861930 | Danio rerio NP_956745 | Batrachochytrium dendrobatidis 29998 | Cyanidioschyzon meroale CMT305C | Canis familiaris XP_532053 |
| Colwellia psychrerythraea YP_270132 | Daphnia pulex 304871 | Bigellowiela natans 46835 | Cyanophora paradoxa 53670 | Canis familiaris XP_544555 |
| Compsopogon sp. CAMPEP_0184681104 | Dolichomastix sp. CAMPEP0170141300 | Branchiostoma floridae 123555 | Danio rerio NP_001004603 | Canis familiaris XP_866797 |
| Compsopogon sp. CAMPEP0184687636 | Dunaliella sp. CAMPEP0202340754 | Branchiostoma floridae 287613 | Daphnia pulex 187509 | Ceratium fusus CAMPEP0199542192 |
| Crustomastix sp. CAMPEP0183809524+0183793952 | Ectocarpus siliculosus CBN77982 | Burkholderia cenocepacia YP_002234614 | Dolichomastix sp. CAMPEP0170145206 | Colwellia psychrerythraea YP_267142 |
| Cryptomonas sp. CAMPEP0172164204 | Erythrobacter sp. NAP1 ZP_01038893 | Calliarthron sp. SRR090438.211357 | Dunaliella sp. CAMPEP0187390102 | Colwellia psychrerythraea YP_267939 |
| Cyanidioschyzon merolae CML055C | Fragilariopsis cylindrus 262171 | Canis familiaris XP_537995 | Ectocarpus siliculosus CBN78379 | Crypthecodinium cohnii CAMPEP0193925118 |
| Cyanidioschyzon merolae CMQ255C | Francisella philomiragia WP_044525538 | Canis familiaris XP_540124 | Emiliania huxleyi 124858 | Danio rerio NP_001071019 |
| Cyanophora paradoxa 10649 | Galdieria sulphuraria Gs05180 | Colwellia psychrerythraea YP_267220 | Erythrobacter sp. NAP1 ZP_01040056 | Danio rerio NP_955905 |
| Cyanophora paradoxa 37037 | Gallus gallus NP_001025885 | Colwellia psychrerythraea YP_271275 | Fragilariopsis cylindrus 207847 | Danio rerio XP_001922598 |
| Daphnia pulex EFX86149 | Gephyrocapsa oceanica CAMPEP0188168580 | Crustomastix sp. CAMPEP0183819616 | Frankia sp. ZP_09173113 | Daphnia pulex 214469 |
| Dictyostelium discoideum CAA39077 | Gloeobacter violaceus NP_923997 | Crypthecodinium cohnii CAMPEP0193909028 | Galdieria sulphuraria Gs30780 | Ectocarpus siliculosus CBJ27497 |
| Dolichomastix sp. CAMPEP0170144458+0170142850 | Gloeochaete witrockiana CAMPEP0194005992 | Cryptomonas sp. CAMPEP0172177232 | Gallus gallus NP_001013413 | Ectocarpus siliculosus CBJ32649 |
| Dunaliella sp. CAMPEP_0202353736 | Guillardia theta 68151 | Cyanidioschyzon merolae CML214C | Gloeobacter violaceus NP_925879 | Ectocarpus siliculosus CBJ48602 |
| Ectocarpus siliculosus CBJ30744 | Hemiselmis sp. CAMPEP0169471182 | Cyanophora paradoxa 6658 | Gloeochaete witrockiana CAMPEP0194017662 | Emiliania huxleyi 434747 |
| Ectocarpus siliculosus CBN78778 | Heterosigma akashiwo CAMPEP0188666620 | Danio rerio NP_001009884 | Goniomonas pacifica CAMPEP0188451540 | Fragilariopsis cylindrus 182884 |
| Emiliania huxleyi 422007 | Chattonella subsalsa CAMPEP0187171642 | Danio rerio XP_001334671 | Guilardia theta 68321 | Gallus gallus XP_001234945 |
| Emiliania huxleyi 463837 | Chlorobium tepidum NP_661946 | Daphnia pulex 187511 | Hemiselmis sp. CAMPEP0169450010 | Gloeochaete witrockiana CAMPEP0193992320 |
| Erythrobacter sp. NAP1 ZP_01039621 | Chrysochromulina polylepis CAMPEP0193798526 | Dictyostelium discoideum XP_643196 | Heterosigma akashiwo CAMPEP0188652810 | Goniomonas pacifica CAMPEP0188538666 |
| Fragilariopsis cylindrus 169332 | Isochrysis sp. CCMP1324 CAMPEP0188834298 | Dolichomastix sp. CAMPEP0170132668 | Chattonella subsalsa CAMPEP0187156594 | Guillaria theta 84137 |
| Fragilariopsis cylindrus 264682 | Karenia brevis CAMPEP0189335222 | Dunaliella sp. CAMPEP0187375806 | Chlamydomonas reinhardtii 458140 | Hemiselmis sp. CAMPEP 0169481540 |
| Gallus gallus NP_001039306 | Karlodinium micrum CAMPEP0200825514 | Ectocarpus siliculosus CBJ48729 | Chlorobium tepidum NP_662005 | Chromera velia 2806.t1 |
| Gloeobacter violaceus NP_924715 | Laccaria bicolor 290764 | Ectocarpus siliculosus CBN76155 | Chromera velia 4809.t1 | Chrysochromulina polylepis CAMPEP0193788762 |
| Gloeochaete witrockiana CAMPEP0194002802 | Lotharella globosa CAMPEP0190169400 | Emiliania huxleyi 204139 | Chrysochromulina polylepis CAMPEP0193719170 | Isochrysis sp. CCMP1324 CAMPEP0188830998 |

Guilllardia theta XP_005822059
Guilllardia theta XP_005822387
Halanaerobium hydrogeniformans WP_013406262
Hemiselmis sp. CAMPEP0169440248
Hemiselmis sp. CAMPEP0169440778
Heterosigma akashiwo CAMPEP0188660984
Chattonella subsalsa CAMPEP0187152510
Chlamydomonas reinhardti 195255
Chromera velia 10392.t1
Chrysochromulina polylepis CAMPEP0193716026
Isochrysis sp. CCMP1324 CAMPEP0188811158
Isochrysis sp. CCMP1324 CAMPEP0188835724
Karenia brevis CAMPEP0189322926
Karlodinium micrum CAMPEP0200798538
Karlodinium micrum CAMPEP0200802162
Laccaria bicolor 184504
Laccaria bicolor 294748
Lankesteria sp. CAMPEP0113851332
Leishmania braziliensis XP_001563662
Leishmania infantum XP_001464549
Lingulodinium polyedra CAMPEP0190064066
Lotharella globosa CAMPEP0190165330
Lotharella globosa CAMPEP0190180660
Methylobacterium radiotolerans YP_001753261
Micromonas pusilla 104797
Monodelphis domestica XP_001369265
Monodelphis domestica XP_001371124
Monosiga brevicolis 17284
Monosiga brevicolis 37518
Mucor circinelloides 136456
Mucor circinelloides 160822
Mycobacterium tuberculosis ZP_02550712
Naegleria gruberi 59636
Neisseria meningitidis YP_003082542
Nematostella vectensis 189113
Nematostella vectensis gw.270.5.1
Neobodo saliens BAF95078
Neurospora crassa NCU02677T0
Neurospora crassa NCU08287T0
Ostreococcus lucimarinus 9901+19640
Oxyrrhis marina CAMPEP0190331566
Parabodo caudatus BAF95073
Paulinella chromatophora ACB43225
Pavlova sp. CCMP459 CAMPEP0190500212
Pelagomonas calceolata CAMPEP0199689514
Pelagomonas calceolata CAMPEP0199697836
Perkinsus marinus XP_002784057
Phaeodactylum tricornutum XP_002183539
Phaeodactylum tricornutum XP_002185520
Phanerochaete chrysosporium 124453
Methylobacterium radiotolerans YP_001753324
Micromonas pusilla 79415
Monodelphis domestica XP_001362923
Monosiga brevicolis 11183
Neisseria meningitidis YP_003082740
Nematostella vectensis 172052
Neurospora crassa 7115
Oceanicola sp. ZP_09515012
Ochromonas sp. CCMP1393 CAMPEP0190270026
Ostreococcus lucimarinus 26950
Oxyrrhis marina CAMPEP0190372354
Paenibacillus dendritiformis ZP_09674768
Pavlova sp. CCMP459 CAMPEP0190518138
Pelagomonas calceolata CAMPEP0199696362
Peridinium aciculiferum CAMPEP0190614724
Phaeodactylum tricornutum XP_002178853
Phanerochaete chrysosporium 132875
Phykomyces blakesleeanus 133556
Physcomitrella patens 112045
Phytophthora infestans XP_002896633
Phytophthora ramorum 71379
Phytophthora sojae 109558
Pleurochrysis carterae CAMPEP0190752624
Populus trichcarpa 830251
Proboscia alata CAMPEP0200157872
Prochlorococcus marinus YP_001549897
Prorocentrum minimum CAMPEP0191087538
Pyramimonas sp. CAMPEP0191475010
Rattus norvegicus NP_067588
Rhodella maculata CAMPEP0191517528
Rhodobacter sphaeroides YP_353803
Saccharomyces cerevisiae NP_011882
Shewanella halifaxensis YP_001676288
Sorangium cellulosum KYF79744
Spirochaeta thermophila WP_013314118
Sulfolobus solfataricus NP_342157
Symbiodinium microadriaticum OLQ00940
Symbiodinium sp. Mp CAMPEP_0192604678
Tetraselmis sp. CAMPEP0200913508
Thalassiosira pseudonana XP_002291698
Thermaerobacter subterraneus ZP_11320829
Thermobaculum terrenum WP_012874271
Thermosynechococcus elongatus NP_681156
Trichodesmium erythraeum YP_720644
Trichoplax adherens 30108
Vaucheria litorea CAMPEP0199148394
Vibrio vulnificus NP_760296
Vitrella brassicaformis CEM03321
Emiliania huxleyi 53510
Erythrobacter sp. NAP1 ZP_01040708
Eutreptiella gymnastica-like CAMPEP0200409666
Eutreptiella gymnastica-like CCMP1594 CAMPEP020042[illegible]
Fervidobacterium nodosum YP_001410216
Fragilariopsis cylindrus 190753
Fragilariopsis cylindrus 268473
Galdieria sulphuraria Gs00120.1
Gallus gallus NP_990241
Gephyrocapsa oceanica CAMPEP0188185824
Giardia lamblia XP_001709979
Gloeobacter violaceus NP_926047
Gloeochaete witrockiana CAMPEP0194021016
Goniomonas pacifica CAMPEP0188507012
Gordonia sp. YP_006669506
Guillardia theta 117281
Halobacterium sp. NP_395811
Hemiselmis sp. CAMPEP0169432848
Heterosigma akashiwo CAMPEP0188659862
Chattonella subsalsa CAMPEP0187174736
Chlamydomonas reinhardtii 188762
Chlamydomonas reinhardtii 31
Chlorobium tepidum NP_662003
Chlorobium tepidum NP_662679
Chromera velia 22655.t1
Chrysochromulina polylepis CAMPEP0193786714
Isochrysis sp. CCMP1324 CAMPEP0188809732
Karenia brevis CAMPEP0189442152
Karenia brevis CAMPEP0189493900
Karlodinium micrum CAMPEP0200767534
Karlodinium micrum CAMPEP0200795676
Laccaria bicolor 251532
Laccaria bicolor 294748
Leishmania braziliensis XP_001563658
Leishmania infantum XP_001464542
Lingulodinium polyedra CAMPEP0190024896
Lotharella globosa CAMPEP0190170490
Methylobacterium radiotolerans YP_001753702
Methylobacterium radiotolerans YP_001754995
Microminas pusilla 98407
Micromonas pusilla 55476
Monodelphis domestica XP_001371124
Monodelphis domestica XP_001379029
Monosiga brevicolis 11184
Monosiga brevicolis 37518
Neisseria meningitidis YP_003083549
Nematostella vectensis 182282
Nematostella vectensis 189113
Neurospora crassa 1471
Neurospora crassa 7228
Isochrysis sp. CCMP1324 CAMPEP0188815466
Isosphaera pallida YP_004180023
Karlodinium micrum CAMPEP0200846968
Laccaria bicolor 159434
Leishmania braziliensis XP_001563662
Leishmania infantum XP_001464549
Lentisphaera a raneosa ZP_01875994
Leptonema illini ZP_09660323
Lingulodinium polyedra CAMPEP0189961030
Lotharella globosa CAMPEP0190174816
Mesodinium sp. CAMPEP0116906476
Methylobacterium radiotolerans YP_001753303
Micromonas pusilla 64373
Monodelphis domestica XP_001364691
Monosiga brevicolis 11193
Neisseria meningitidis YP_003082294
Nematostella vectensis 173898
Neurospora crassa 2319 Aspergillus oryzae XP_0018209[illegible]
Ochromonas sp. CCMP1393 CAMPEP0190297066
Ostreococcus lucimarinus 19853
Oxyrrhis marina CAMPEP0190380228
Paulinella chromatophora contig00353
Pavlova sp. CCMP459 CAMPEP0190508342
Pelagomonas calceolata CAMPEP0199678114
Phaeodactylum tricornutum XP_002185721
Phanerochaete chrysosporium 127677
Phycomyces blakesleeanus 182982
Physcomitrella patens 98439
Phytophthora infestans XP_002999029
Phytophthora ramorum 72088
Phytophthora sojae 108409
Picocystis sp. CAMPEP0190741344
Plesiocystis pacifica ZP_01911475
Pleurochrysis carterae CAMPEP0190802484
Populus trichocarpa 228730
Populus trichocarpa 579188
Porphyra yezoensis AV435201
Porphyridium cruentum HS637120
Proboscia alata CAMPEP0200184052
Prochlorococcus marinus YP_001551729
Prorocentrum minimum CAMPEP0191110192
Prymnesium parvum CAMPEP0191256308
Pseudomonas sp. ZP_10666004
Pterosperma sp. CAMPEP0197868708
Pycnococcus sp. CAMPEP0205958814
Pyramimonas sp. CAMPEP0191478448
Rattus norvegicus NP_037289
Rhodella maculata CAMPEP0191517038
Rhodobacter sphaeroides YP_354294
Rhodomonas sp. CCMP768_CAMPEP0191544722
Karlodinium micrum CAMPEP0200755204
Karlodinium micrum CAMPEP0200801830
Laccaria bicolor 176615
Laccaria bicolor 305021
Leishmania braziliensis XP_001568250
Leishmania infantum XP_001468968
Lotharella globosa CAMPEP0190152330
Methylobacterium radiotolerans YP_001755148
Micromonas pusilla 58240
Micromonas pusilla 70593
Monodelphis domestica XP_001370052
Monodelphis domestica XP_001377520
Monodelphis domestica XP_001380383
Naegleria gruberi 74735
Nematostella vectensis 211198
Nematostella vectensis 224716
Nematostella vectensis 241846
Neurospora crassa 1202
Neurospora crassa 1819
Neurospora crassa 2046
Neurospora crassa 9095
Ochromonas sp. CCMP1393 CAMPEP0190296750
Oxyrrhis marina CAMPEP0190330902
Paraphysomonas imperforata CAMPEP0190475706
Paulinella chromatophora ACB42823
Pavlova sp. CCMP459 CAMPEP0190514400
Peridinium aciculiferum CAMPEP0190619828
Perkinsus marinus XP_002768228
Phaeodactylum tricornutum XP_002182650
Phaeodactylum tricornutum XP_002184908
Phanerochaete chrysosporium 124436
Phanerochaete chrysosporium 132538
Phanerochaete chrysosporium 29874
Phycomyces blakesleeanus 133419
Phycomyces blakesleeanus 171705
Physcomitrella patens 158155
Physcomitrella patens 234776
Phytophthora infestans XP_002894702
Phytophthora ramorum 75495
Phytophthora sojae 141130
Pleurochrysis carterae CAMPEP0190754232
Populus trichocarpa 572088
Populus trichocarpa 597276
Proboscia alata_CAMPEP0200177464
Prochlorococcus marinus YP_001551702
Prymnesium parvum CAMPEP0191201332
Pteridomonas danica CAMPEP0193803406
Rattus norvegicus BAA13183
Rattus norvegicus EDL87778
Rattus norvegicus NP_001041650

Phanerochaete chrysosporium 5965
Phycomyces blakesleeanus 124538
Phycomyces blakesleeanus 74982
Physcomitrella patens 180534
Phytophthora infestans XP_002902242
Phytophthora infestans XP_002906192
Phytophthora ramorum 46151
Phytophthora ramorum 71989
Phytophthora sojae 109206
Phytophthora sojae 109425
Plasmodium falciparum XP_001349809
Pleurochrysis carterae CAMPEP0190806706
Pleurochrysis carterae CAMPEP0190807314
Populus trichocarpa 229687
Proboscia alata CAMPEP0200141504
Proboscia alata CAMPEP0200149434
Prochlorococcus marinus YP_001550827
Prymnesium parvum CAMPEP0191199356
Prymnesium parvum CAMPEP0191199560
Pyramimonas sp. CAMPEP0191508516+0191501876
Rattus norvegicus NP_001099180
Rattus norvegicus NP_058768
Rhodobacter sphaeroides YP_353897
Rhodomonas sp. CCMP768 CAMPEP0191546976
Rhodopirellula baltica NP_870325
Rhodosorus sp. CAMPEP0184741054
Rhodosorus sp. CAMPEP0184753238
Saccharomyces cerevisiae YJL130C
Saccharomyces cerevisiae YJR109C
Salpingoeca rosetta EGD76319
Shewanella halifaxensis YP_001675367
Sulfolobus solfataricus NP_342159
Tetraselmis sp. CAMPEP0200903730
Thalassiosira pseudonana XP_002289336
Thalassiosira pseudonana XP_002292735
Theileria annulata XP_954765
Theileria parva XP_763067
Thermosynechococcus elongatus NP_683122
Thraustochytrium sp. LLF1b CAMPEP0193131092
Thraustochytrium sp. LLF1b CAMPEP0193133550
Toxoplasma gondii AAL27793
Trichodesmium erythraeum YP_722523
Trichoplax adherens 29177
Trypanosoma brucei CBH11387
Vaucheria litorea CAMPEP0199153208
Vaucheria litorea CAMPEP0199157910
Vibrio vulnificus CMCP6
Vitrella brassicaformis CEM14616

Ochromonas sp. CCMP1393 CAMPEP0190276052
Ostreococcus lucimarinus 13232
Ostreococcus lucimarinus 28754
Oxyrrhis marina CAMPEP0190328430
Paulinella chromatophora ACB42582
Pavlova sp. CCMP459 CAMPEP0190508088
Pelagomonas calceolata CAMPEP0199691512
Peridinium aciculiferum CAMPEP0190620402
Phaeodactylum tricornutum XP_002184453
Phanerochaete chrysosporium 125524
Phanerochaete chrysosporium 5965
Phycomyces blakesleeanus 113947
Phycomyces blakesleeanus 184165
Physcomitrella patens 146128
Physcomitrella patens 169569
Phytophthora infestans XP_002904952
Phytophthora ramorum 49061
Phytophthora ramorum 95147
Phytophthora sojae 109166
Phytophtjora sojae 155409
Picocystis sp. CAMPEP0190745440
Plasmodium falciparum XP_001350162
Pleurochrysis carterae CAMPEP0190760360
Populus trichocarpa 206001
Populus trichocarpa 550113
Populus trichocarpa 794796
Porphyridium cruentum HS970446+HS695246
Proboscia alata CAMPEP0200159054
Prochlorococcus marinus YP_001551191
Prorocentrum minimum CAMPEP0191100692
Prymnesium parvum CAMPEP0191200986
Pyramimonas sp. CAMPEP0191494844
Rattus norvegicus CAA26007
Rattus norvegicus EDM02939
Rhodella maculata CAMPEP0191514062
Rhodobacter sphaeroides YP_352060
Rhodomonas sp. CCMP768 CAMPEP0191587138
Rhodopirellula baltica NP_867004
Rhodosorus sp. CAMPEP0184744098
Roseiflexus castenholzii YP_001433145
Saccharomyces cerevisiae NP_012405
Saccharomyces cerevisiae NP_012447
Scrippsiella trochoidea CAMPEP0191998236
Shewanella halifaxensis YP_001672921
Shewanella halifaxensis YP_001675259
Sorangium cellulosum WP_012239766
Sulfolobus solfataricus NP_342369
Symbiodinium sp. Mp CAMPEP0192595932
Tetraselmis sp. CAMPEP0200904340
Thalassiosira pseudonana XP_002286586

Rhodopirellula baltica NP_868287
Rhodosorus CAMPEP0184744750
Saccharomyces cerevisiae NP_014583
Scrippsiella trochoidea CAMPEP0192006414
Shewanella halifaxensis YP_001676289
Sorangium cellulosum WP_012237678
Sulfolobus solfataricus NP_342156
Symbiodinium sp. Mp CAMPEP0192576618
Terriglobus roseus YP_006421345
Tetraselmis sp. CAMPEP0200903406
Thalassiosira pseudonana XP_002295518
Thermoplasma acidophilum NP_394121
Thermosynechococcus elongatus NP_681501
Thraustochytrium sp. LLF1b CAMPEP0193136054
Trichodesmium erythraeum YP_720757
Trichoplax adherens 21771
Trypanosoma brucei XP_845050
Trypanosoma cruzi XP_805210
Vaucheria litorea CAMPEP0199152120
Vibrio vulnificus NP_760295
Vitrella brassicaformis 1163.t1

Rhodobacter sphaeroides YP_352689
Rhodobacter sphaeroides YP_352971
Rhodomonas sp. CCMP768 CAMPEP0191588228
Saccharomyces cerevisiae NP_015214
Scrippsiella trochoidea CAMPEP0192095212
Selaginella moellendorffii 73545_e_gw1.0.671.1
Shewanella halifaxensis YP_001674029
Sorangium cellulosum WP_012232678
Sorangium cellulosum WP_012237032
Sulfolobus solfataricus NP_344052
Thalassiosira pseudonana XP_002296117
Thraustochytrium sp. LLF1b CAMPEP0193130326
Trichoplax adherens 52723
Vibrio vulnificus NP_761202
Vibrio vulnificus NP_936803
Vitrella brassicaformis 27786.t1
Vitrella brassicaformis 8209.t1

Thalassiosira pseudonana XP_002291441
Theileria annulata XP_954289
Theileria parva XP_765996
Thermococcuskodakarensis YP_183284
Thermoplasma acidophilum NP_394049
Thermoplasma acidophilum NP_394786
Thermosynechococcus elongatus NP_681897
Thraustochytrium sp. LLF1b CAMPEP0193140552
Trichodesmium erythraeum YP_721117
Trichomonas vaginalis XP_001301097
Trichoplax adherens 29177
Trypanosoma brucei XP_845052
Vaucheria litorea CAMPEP0199158548
Vibrio vulnificus NP_760377
Vibrio vulnificus NP_760378
Vitrella brassicaformis 781.t1
